# Pan-immune-inflammation value and sleep disturbances in rheumatoid arthritis: A population-based NHANES study

**DOI:** 10.1097/MD.0000000000051348

**Published:** 2026-10-23

**Authors:** Yuting Xiao, Jiayou Zhang, Shaoling Han, Lijuan Chen, Junhui Liu, Jing Wu, Jing Su

**Affiliations:** a Department of Hematology and Rheumatology, The Second Affiliated Hospital of Chengdu Medical College, Nuclear Industry 416 Hospital, Chengdu, Sichuan, China.

**Keywords:** NHANES, pan-immune-inflammation value, rheumatoid arthritis, trouble sleeping

## Abstract

The present study sought to assess the individual and synergistic effects of pan-immunoinflammatory value (PIV) and trouble sleeping on rheumatoid arthritis (RA). We used data from the National Health and Nutrition Examination Survey from 2005 to 2020. PIV was computed as: (neutrophil count × platelet count × monocyte count) /lymphocyte count. Trouble sleeping was assessed using a single self-reported question (National Health and Nutrition Examination Survey item SLQ050), while RA case ascertainment relied on self-reported diagnosis by a physician. The associations between PIV and sleep-related RA susceptibility were interrogated through multivariable logistic regression models, with restricted cubic splines deployed to unmask potential nonlinear relationships. Effect modification was further evaluated via stratified analyses across demographic and clinical subgroups. The analytical cohort comprised 27,398 participants (2032 RA cases). In fully adjusted models, elevated PIV demonstrated a 14.9% increased RA risk in the highest versus lowest PIV quartile (Q4 vs Q1) (OR = 1.149, 95% CI:1.007−1.312), while trouble sleeping independently conferred 59.8% higher odds (OR = 1.598, 95% CI:1.449−1.761). Interaction analysis revealed that the association between PIV and RA was modulated by trouble sleeping. This study explores the association between elevated PIV, trouble sleeping, and RA. However, due to the cross-sectional nature of the data, causality cannot be inferred. The findings suggest a correlation between PIV and trouble sleeping with RA risk, but further longitudinal studies are required to establish causal relationships.

## 1. Introduction

Rheumatoid arthritis (RA) is a chronic inflammatory disease that typically affects joints, leading to progressive damage. It is most common in adults over 30 years of age, with a higher prevalence in women.^[1]^ The disease contributes significantly to physical disability and has a considerable impact on patients’ quality of life. RA mainly affects small, symmetrical peripheral joints; however, it can also affect large peripheral joints and other organs and tissues, including the lungs, cardiovascular system, and nervous system.^[2]^ The hallmark pathological characteristic of RA is aberrant proliferative inflammation of synovial tissue. This chronic synovitis instigates progressive degradation of the articular cartilage and destruction of the bone matrix through inflammatory cell infiltration and release of pro-inflammatory factors, resulting in irreversible structural joint deformities and loss of function.^[3,4]^ RA not only has an impact on physical function and reduces the social participation of patients, but also places a financial burden on the families of patients.^[5]^ However, the progression and prognosis of RA can be managed and modified, with effective treatment and lifestyle interventions playing a key role.^[6]^

Recent studies have revealed that a novel immune-inflammation assessment index, pan-immune-inflammation value (PIV), based on the integration of multidimensional blood parameters, has been established as a key biomarker for the systematic assessment of immune-inflammatory status. The quantification of these parameters is achieved through the implementation of a standardized formula, which incorporates the neutrophil count, monocyte count, platelet count, and lymphocyte count.^[7]^ This approach circumvents the limitations imposed by conventional single inflammatory indicators, thereby facilitating a more precise reflection of the dynamic fluctuations in the body’s immune-inflammation equilibrium. PIV, as an integrated measure of systemic inflammation, is used to assess the intensity of immune activation in various diseases, including cancer, infections, and autoimmune disorders.^[8]^ In the context of RA, high PIV levels reflect heightened systemic inflammation, which is closely associated with disease progression.^[9–12]^ While this study demonstrates that elevated PIV is linked to increased RA risk, it is important to recognize that PIV may serve as a tool for identifying individuals at higher risk, potentially guiding early intervention strategies.^[13]^ However, further research is needed to evaluate whether reducing PIV levels can directly prevent or modify the course of RA.^[14,15]^

Sleep is a cyclical, spontaneous, and reversible phenomenon that occupies one-third of the human lifespan. A night of adequate sleep can contribute to physical recovery, emotional regulation, and influences metabolism and brain function.^[16]^ Extended periods of sleep, exceeding 7 hours, have been demonstrated to be more conducive to optimal health.^[17]^ Adequate sleep is associated with a robust immune system. Conversely, sleep disorders have been demonstrated to induce immune system dysfunction and elevate the probability of developing various diseases. Sleep disorders are an underrecognized feature of RA.^[18,19]^ Shorter sleep duration at night is a common occurrence in RA, and sleep deprivation elevates the cardiovascular risk in early RA.^[20]^ The heightened sensitivity of the immune system to sleep, as well as the regulatory influence of sleep on immunity and inflammation, has been well documented. Consequently, enhancing sleep quality has the potential to reduce inflammation and promote overall health.^[21]^

Autoimmune diseases have been shown to increase inflammatory factors, and early symptoms may include sleep disorders. Patients diagnosed with autoimmune diseases require immunotherapy, a therapeutic modality that has the capacity to suppress inflammation and enhance sleep quality.^[22]^ Consequently, there is a possibility for an interaction between PIV and trouble sleeping, which can result in RA. To date, the academic community has yet to elucidate the mechanism of interaction between PIV and trouble sleeping in the pathogenesis of RA. Although the present study identifies significant associations between PIV, trouble sleeping, and RA, it is important to note that the cross-sectional design of the study limits the ability to draw conclusions about causality. Our findings suggest correlations, but further longitudinal or interventional studies are needed to clarify causal mechanisms. This study is pioneering in its systematic exploration of the association between the 3, based on the National Health and Nutrition Examination Survey (NHANES) database. This finding provides a novel intervention target for RA prevention: a multidimensional strategy (anti-inflammatory treatment combined with cognitive behavioral therapy to improve sleep) may break the vicious cycle of “inflammation-sleep disorder-joint destruction.”

## 2. Methods

### 2.1. Data selection and study design

The data for this study were obtained from the publicly available database of the 2005 to 2020 cycle of the NHANES. All participants provided informed consent, with data publicly available via the Centers for Disease Control and Prevention.

The initial study sample comprised 76,496 respondents, and the final analysis cohort was derived through systematic data cleaning. Firstly, subjects with missing data concerning RA diagnosis, PIV-related blood parameters (neutrophil, monocyte, lymphocyte, and platelet counts), and trouble sleeping assessment were excluded from the study. Secondly, subjects with incomplete information on covariates, including demographic characteristics, clinical indicators, and lifestyle factors, were excluded based on the American Heart Association. Samples with incomplete information on demographic characteristics, clinical indicators, and covariates such as lifestyle were excluded. Participants aged under 20 years were excluded. This resulted in the enrollment of 27,398 eligible participants, including 2032 patients with clinically diagnosed RA (Fig. 1).

**Figure 1.**
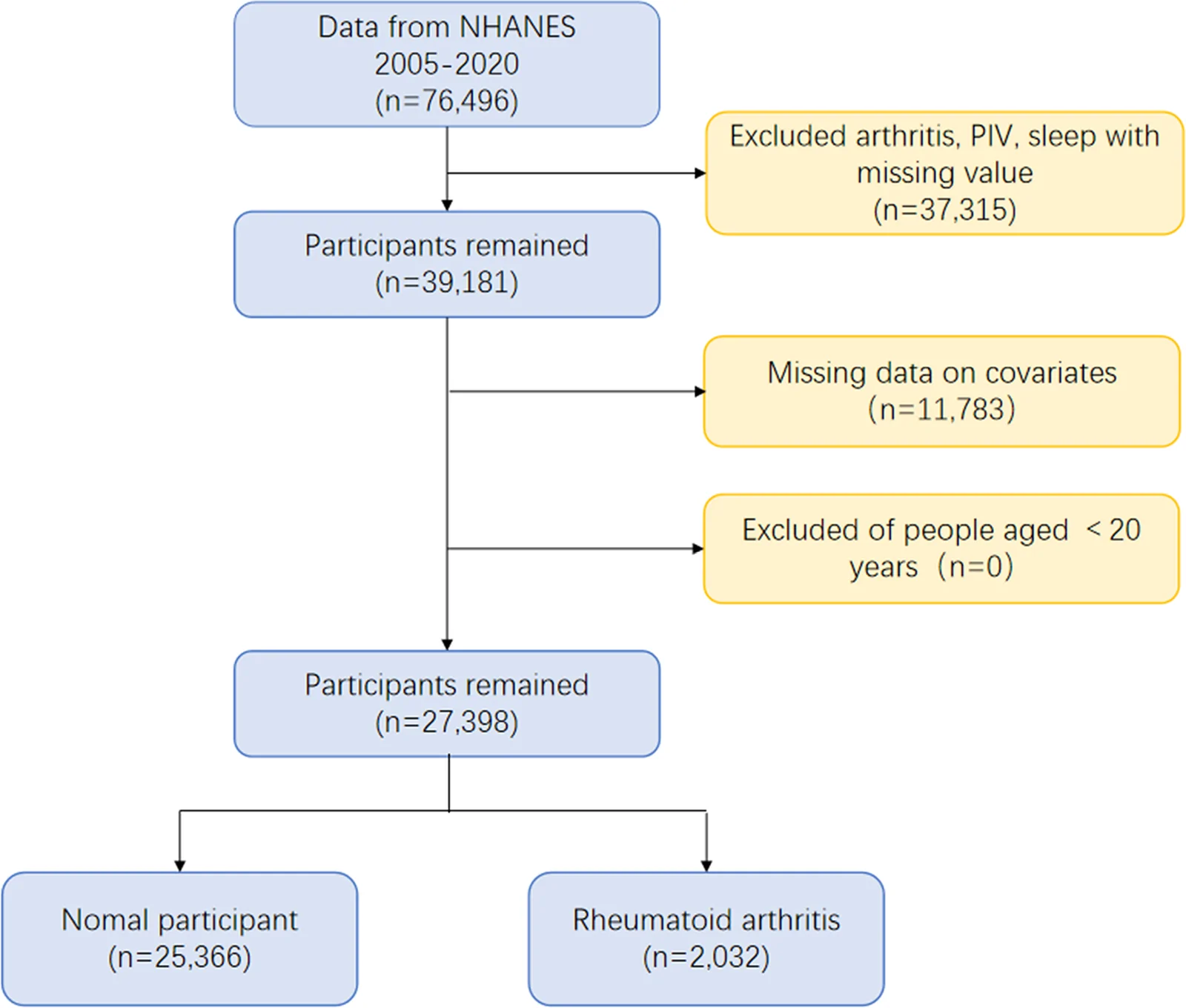
Study design flowchart. NHANES = National Health and Nutrition Examination Survey, PIV = pan-immune-inflammation value.

### 2.2. Exposure and outcome definitions

#### 2.2.1. Assessment of PIV

The value of PIV is derived through calculation, using the following equation: PIV = neutrophil count × platelet count × monocyte count/lymphocyte count.^[7]^

#### 2.2.2. Assessment of trouble sleeping

Trouble sleeping was ascertained using a single self-reported item from the NHANES Sleep Disorder Questionnaire (variable SLQ050): “Have you ever told a doctor or other health professional that you have trouble sleeping?” A “yes” answer was classified as trouble sleeping, while a “no” answer was classified as no trouble sleeping.^[23]^

#### 2.2.3. Definition of RA

RA cases are identified from self-diagnosis by a physician on a medical condition questionnaire. Confirmation of “Has a doctor or other healthcare professional ever told you that you have arthritis?” Participants who answered yes went on to answer “What type of arthritis was diagnosed?” This follow-up item was confirmed. Respondents who explicitly reported “RA” were categorized as RA cases.^[24]^

### 2.3. Covariate assessment

To comprehensively evaluate factors associated with RA, we accounted for demographic characteristics including age (<40, 40−59, ≥60), sex (male, female), race (Mexican American, Other Hispanic, Non‐Hispanic White, Non‐Hispanic Black, Other races), education level (<9th Grade, 9–11th Grade, High School Grad/GED or Equivalent, Some College or AA degree, College Graduate or Above), marital status (Married, Widowed, Divorced, Separated, Never married, Living with partner), and poverty-to-income ratio ([PIR] < 1, 1–3, >3). We additionally considered body mass index (BMI < 25, 25–30, >30), smoking status, and alcohol consumption status. Assessed comorbidities included hypertension, hyperlipidemia, diabetes mellitus, and coronary heart disease (all categorized as yes/no).

### 2.4. Statistical analysis

PIV was categorized into quartiles (Q1–Q4) from lowest to highest values. Continuous variables are presented as mean ± standard error (SE), while categorical variables are reported as frequencies and percentages (%). Baseline characteristics were compared using chi-square tests for categorical variables and Student’s t tests for continuous variables. We employed multiple logistic regression to examine the association between PIV, trouble sleeping, and RA risk across 3 progressively adjusted models. Model 1 is a crude model that has not undergone adjustment. Model 2 is a gender-age-adjusted model. Model 3 has undergone full adjustment to account for all available covariates. In order to comprehend the potential non-linear association between PIV and RA, a restricted cubic spline (RCS) analysis was conducted. Subsequently, subgroup analyses and interaction analyses were performed, with the following factors considered: sex, age, race, PIR, BMI, hypertension, diabetes, hyperlipidemia, and coronary heart disease.

All statistical analyses were performed using IBM SPSS Statistics (version 27) and R software (version 4.5.0). All hypothesis tests were conducted as 2-sided analyses, with a predetermined significance threshold of *P* < .05.

## 3. Results

### 3.1. Basic characteristics of participants

The study cohort comprised 27,398 participants, with 7.42% (n = 2032) meeting criteria for RA. The overall population had a mean age of 50.90 years, consisting of 13,263 males (48.41%) and 14,135 females (51.59%). Comparative analysis revealed that the RA group were older than non-RA counterparts (62.51 ± 0.30 vs 49.97 ± 0.11 years, *P* < .001). Demographic and clinical characteristics differed significantly between groups (all *P* < .05). Lifestyle factors including current smoking status and higher BMI were more prevalent in RA participants. Comorbidity burden was substantially greater in the RA group, with higher rates of diabetes (22.19% vs 13.88%), hypertension (59.94% vs 36.91%), hyperlipidemia (52.36% vs 36.71%), and coronary heart disease (8.56% vs 4.23%). Trouble sleeping was reported more frequently among RA participants (41.29% vs 26.53%). PIV demonstrated significant elevation in the RA group compared to controls (353.21 ± 8.13 vs 307.60 ± 1.59, *P* < .001), suggesting a robust association between systemic inflammation and RA status (Table 1).

**Table 1:** Demographic characteristics in patients with RA.

Characteristic		Overall	Non-RA	RA	*P* value
Age (yr)		50.90 (0.11)	49.97 (0.11)	62.51 (0.30)	<.001
Age, yr, n (%)					<.001
	20–39	8263 (30.16)	8141 (32.09)	122 (6.00)	
	40–59	9279 (33.87)	8671 (34.18)	608 (29.92)	
	≥ 60	9856 (35.97)	8554 (33.72)	1302 (64.07)	
Gender, n (%)					<.001
	Male	13,263 (48.41)	12,448 (49.07)	815 (40.11)	
	Female	14,135 (51.59)	12,918 (50.93)	1217 (59.89)	
Marital status, n (%)					<.001
	Married	15,195 (55.46)	14,067 (55.46)	1128 (55.51)	
	Widowed	3153 (11.51)	2736 (10.79)	417 (20.52)	
	Divorced	3578 (13.06)	3306 (13.03)	272 (13.39)	
	Separated	649 (2.36)	597 (2.35)	52 (2.56)	
	Never married	3349 (12.22)	3238 (12.77)	111 (5.46)	
	Living with partner	1474 (5.38)	1422 (5.61)	52 (2.56)	
Race, n (%)					<.001
	Mexican American	3560 (12.99)	3369 (13.28)	191 (9.40)	
	Other Hispanic	2533 (9.25)	2376 (9.37)	157 (7.73)	
	Non‐Hispanic White	12,316 (44.95)	11,195 (44.13)	1121 (55.17)	
	Non‐Hispanic Black	5924 (21.62)	5469 (21.56)	455 (22.39)	
	Other races	3065 (11.19)	2957 (11.66)	108 (5.31)	
Education, n (%)					<.001
	<9th Grade	2263 (8.26)	2061 (8.13)	202 (9.94)	
	9–11th grade	3378 (12.33)	3107 (12.25)	271 (13.34)	
	High school Grad/GED or Equivalent	6181 (22.56)	5670 (22.35)	511 (25.15)	
	Some College or AA degree	8563 (31.25)	7925 (31.24)	638 (31.40)	
	College graduate or above	7013 (25.60)	6603 (26.03)	410 (20.18)	
PIR, n (%)					<.001
	Below 1	5056 (18.45)	4643 (18.30)	413 (20.32)	
	1–3	11,331 (41.36)	10,453 (41.21)	878 (43.21)	
	Over 3	11,011 (40.19)	10,270 (40.49)	741 (36.47)	
BMI, n (%)					<.001
	Below 25	7446 (27.18)	7036 (27.74)	410 (20.18)	
	25–30	8944 (32.64)	8266 (32.59)	678 (33.37)	
	Over 30	11,008 (40.19)	10,064 (39.68)	944 (46.46)	
Smoking, n (%)					<.001
	Yes	12,144 (44.32)	11,085 (43.70)	1059 (52.12)	
	No	15,254 (55.68)	14,281 (56.30)	973 (47.89)	
Alcohol use, n (%)					<.001
	Yes	20,822 (76.00)	19,400 (76.48)	1422 (69.98)	
	No	6576 (24.00)	5966 (23.52)	610 (30.02)	
Hypertension, n (%)					<.001
	Yes	10,580 (38.62)	9362 (36.91)	1218 (59.94)	
	No	16,818 (61.38)	16,004 (63.09)	814 (40.06)	
Diabetes, n (%)					<.001
	Yes	3971 (14.49)	3520 (13.88)	451 (22.19)	
	No	23,427 (85.51)	21,846 (86.12)	1581 (77.81)	
Hyperlipidemia, n (%)					<.001
	Yes	10,377 (37.88)	9313 (36.71)	1064 (52.36)	
	No	17,021 (62.12)	16,053 (63.29)	968 (47.64)	
coronary heart disease, n (%)					<.001
	Yes	1246 (4.54)	1072 (4.23)	174 (8.56)	
	No	26,152 (95.45)	24,294 (95.77)	1858 (91.44)	
Trouble sleeping, n (%)					<.001
	Yes	7568 (27.62)	6729 (26.53)	839 (41.29)	
	No	19,830 (72.38)	18,637 (73.47)	1193 (58.71)	
PIV		310.98 (1.60)	307.60 (1.59)	353.21 (8.13)	<.001
PIV Quartile					<.001
Quartile 1		6854 (25.02)	6408 (25.26)	446 (21.95)	
Quartile 2		6863 (25.05)	6396 (25.21)	467 (22.98)	
Quartile 3		6858 (25.03)	6349 (25.03)	509 (25.05)	
Quartile 4		6823 (24.90)	6213 (24.49)	610 (30.02)	

BMI = body mass index, PIR = poverty-to-income ratio, PIV = pan-immune-inflammation value, RA = rheumatoid arthritis.

Continuous variables are presented as mean (standard error); categorical variables as n (%).

### 3.2. Logistic regression analysis

PIV was classified into quartiles as follows: group Q1 (PIV < 156.60), group Q2 (156.60 ≤ PIV < 244.83), group Q3 (244.83 ≤ PIV < 382.52), and group Q4 (PIV ≥ 382.52). Multiple logistic regression analyses revealed a positive correlation between elevated PIV scores and an augmented risk of developing RA. This association was significant in all 3 models. In the crude model, Q4 participants demonstrated a 41.1% higher risk of RA compared to Q1 (OR 1.411, 95% CI 1.242−1.602). This association remained significant after full adjustment for demographic, lifestyle, and clinical factors, with Q4 participants maintaining a 14.9% increased RA risk (OR 1.149, 95% CI 1.007−1.312). The persistence of this association through progressive model adjustment suggests an independent relationship between systemic inflammation and RA pathogenesis (Table 2).

**Table 2:** Association between PIV and RA.

Variables	Model 1		Model 2		Model 3	
	OR (95 % CI)	*P*-value	OR (95 % CI)	*P*-value	OR (95 % CI)	*P*-value
Stratified by PIV quartiles						
Q1	ref		ref		ref	
Q2	1.049 (0.917, 1.200)	.485	1.020 (0.890, 1.170)	.775	0.999 (0.870, 1.147)	.986
Q3	1.152 (1.010, 1.314)	.035	1.103 (0.964, 1.262)	.153	1.043 (0.910, 1.195)	.547
Q4	1.411 (1.242, 1.602)	<.001	1.253 (1.100, 1.427)	<.001	1.149 (1.007, 1.312)	.039

No adjustments were made to Model 1. Model 2 was adjusted for sex and age. Model 3 was a fully adjusted model adjusted for age, sex, race, PIR, education level, marriage, BMI, alcohol consumption, smoking, hypertension, diabetes, hyperlipidemia, and coronary heart disease.

OR = Odds ratio, PIV = pan-immune-inflammation value, RA = rheumatoid arthritis.

There is a significant association between trouble sleeping and an increased risk of RA. When the analysis was conducted without controlling for variables, participants who experienced trouble sleeping showed a 94.8% higher RA risk compared to those without sleep problems (OR 1.948, 95% CI 1.776−2.137). Following adjustment for gender and age, individuals experiencing trouble sleeping exhibited a 73.7% higher RA risk (OR 1.737, 95% CI 1.580−1.911). In the fully adjusted model, participants with trouble sleeping exhibited a 59.8% elevated risk of RA (OR 1.598, 95% CI 1.449−1.761) (Table 3).

**Table 3:** Association between trouble sleeping and RA.

Variables	Model 1		Model 2		Model 3	
	OR (95 % CI)	*P*-value	OR (95 % CI)	*P*-value	OR (95 % CI)	*P*-value
Trouble sleeping						
No	ref		ref		ref	
Yes	1.948 (1.776, 2.137)	<.001	1.737 (1.580, 1.911)	<.001	1.598 (1.449, 1.761)	<.001

No adjustments were made to Model 1. Model 2 was adjusted for sex and age. Model 3 was a fully adjusted model adjusted for age, sex, race, PIR, education level, marriage, BMI, alcohol consumption, smoking, hypertension, diabetes, hyperlipidemia, and coronary heart disease.

OR = odds ratio, RA = rheumatoid arthritis.

### 3.3. Restricted cubic splines analysis

RCS analysis showed that PIV was significantly associated with RA risk (*P* Total < .001), but no significant nonlinear trend was detected (*P* for nonlinear = .13), suggesting that a linear model may be sufficient to describe the relationship (Fig. 2).

**Figure 2.**
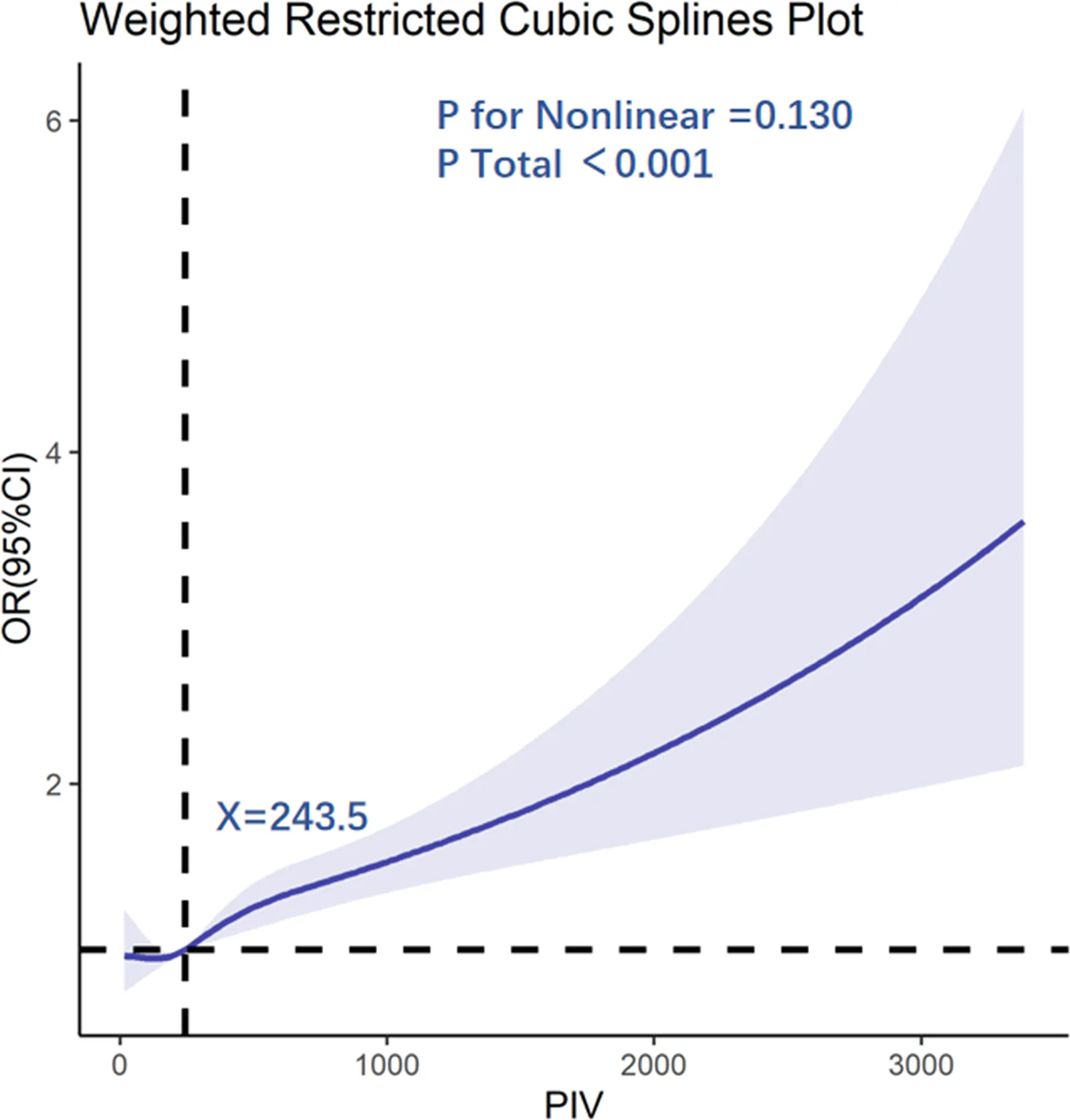
Restricted cubic spline curve assessing the relationship between PIV and RA. PIV = pan-immune-inflammation value, RA = rheumatoid arthritis.

### 3.4. The subgroup analysis and interaction test

Stratified analyses demonstrated differential associations between PIV and RA risk across population subgroups. This investigation found a positive relationship between PIV and RA in subjects under 40 and over 60 years of age. When the data were stratified by BMI, a positive association between PIV and RA was observed in subjects with a BMI <30. When stratified by diabetes and coronary heart disease status, the positive association was observed exclusively in individuals without these conditions (Fig. 3).

**Figure 3.**
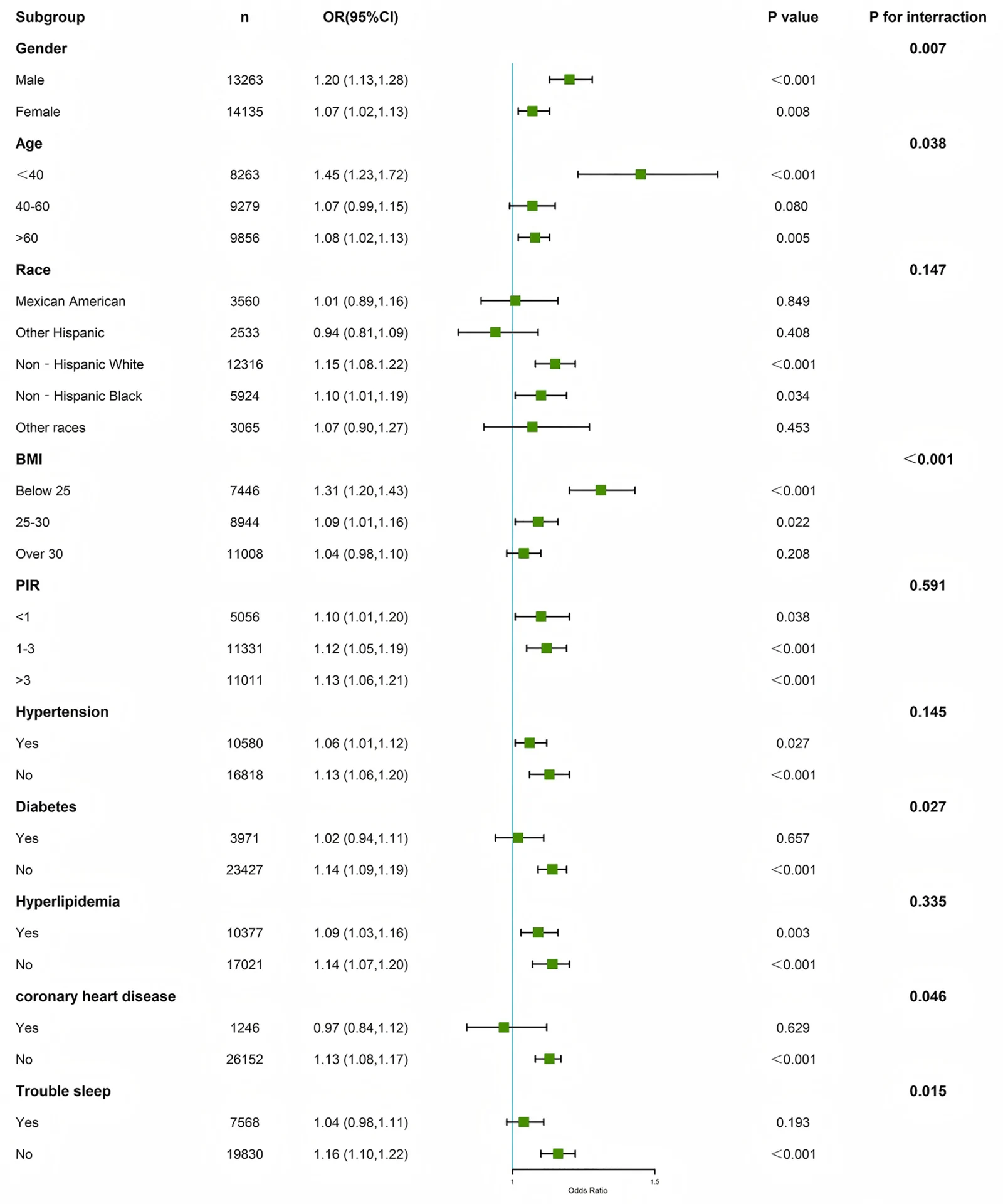
Forest plot of subgroup analysis between PIV and RA. BMI = body mass index, PIV = pan-immune-inflammation value, RA = rheumatoid arthritis.

Stratified analyses revealed significant associations between trouble sleeping and RA across demographic (gender, age, race), anthropometric (BMI), socioeconomic (PIR), and comorbidity subgroups (hypertension, diabetes, coronary heart disease). However, interaction tests demonstrated differential effects: while age, BMI, hypertension, diabetes, hyperlipidemia, and coronary heart disease exhibited significant interaction effects (*P* < .05), no such effect modification was observed for gender, race, or socioeconomic status (PIR) (Fig. 4).

**Figure 4.**
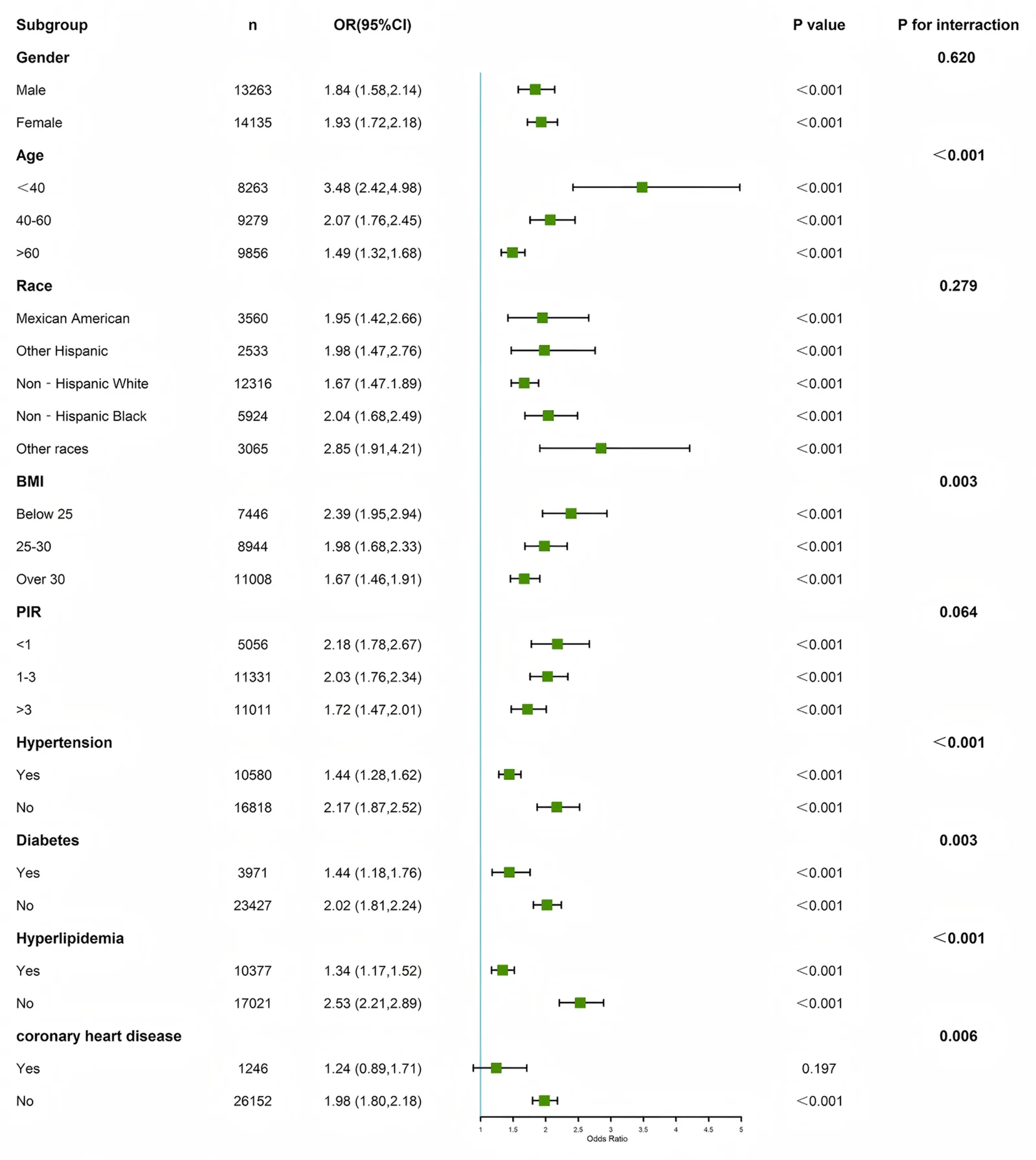
Forest plot of subgroup analysis between trouble sleeping and RA. RA = rheumatoid arthritis.

Interaction analyses of the PIV and RA association revealed distinct modification patterns: clinical comorbidities (diabetes, coronary heart disease), biometric measures (BMI), and trouble sleeping demonstrated significant interaction effects (all *P* < .05), whereas demographic (gender, age), socioeconomic (PIR), and cardiovascular (hypertension) variables showed no modifying capacity (Fig. 3).

## 4. Discussion

This investigation elucidates the complex interplay between PIV, trouble sleeping, and RA pathogenesis through analysis of nationally representative NHANES data. The results suggest a potential association between high PIV and an elevated risk of RA. Trouble sleeping is also independently associated with RA. There is an interaction between PIV and trouble sleeping affecting RA prevalence.

PIV represents a robust integrated measure of systemic inflammation and immune activation. Neutrophils secrete a variety of cytokines that activate other immune cells, and they modulate the adaptive immune response, which promotes the development of cellular immunity by influencing the function of T cells and B cells, while platelets amplify inflammation through granular release of mediators that recruit additional immune cells.^[25]^ The characteristic lymphopenia observed during systemic inflammation reflects both lymphocyte apoptosis and trafficking to inflamed synovial tissue.^[26]^ The etiology of RA remains to be fully elucidated. The pathogenesis of RA involves a multifaceted inflammatory cascade mediated by both soluble mediators and cellular components. Central to this process are proinflammatory cytokines including IL-1, IL-6, IL-17 and tumor necrosis factor-alpha (TNF-α), which interact synergistically with immune cells such as osteoclasts, T cells, and B cells to drive disease progression.^[3,27]^ Notably, dysregulation of IL-6 directly contributes to the osteolytic bone damage characteristic of RA.^[28]^ The synovial tissue exhibits significant macrophage infiltration, with activated macrophages serving as a major source of proinflammatory cytokines.^[29]^ These factors contribute to the process of joint erosion. The accumulation of neutrophils, monocytes, and lymphocytes within the synovium is a hallmark of the inflammatory response, which, if unaddressed, can lead to joint damage. The activation of these immune cells can induce RA. Consequently, the inhibition of cell-induced inflammation has the potential to decelerate the progression of RA.

Sleep plays a critical role in immune regulation. It triggers effective adaptive immunity and generates long-term immune memory. The sleep-immune relationship operates through multiple interconnected pathways, with sleep deprivation or disturbances leading to significant immunological consequences. The neuroendocrine mechanisms linking sleep and immune function are particularly significant. Research has demonstrated a persistent association between sleep disorders and the activation of the sympathetic nervous system and the hypothalamic-pituitary-adrenal axis.^[30,31]^ This dual activation results in the release of corticotropin-releasing hormone, leading to increased wakefulness and decreased sleep quality. The consequences of this neuroendocrine activation are far-reaching, as sleep deprivation has been demonstrated to induce a significant stress response that stimulates the release of various pro-inflammatory cytokines and acute-phase proteins.^[32]^ At the cellular level, sleep disturbances exert profound effects on immune cell function. Current literature indicates that sleep disorders can significantly disrupt the circadian rhythms of T cell proliferation, differentiation, and effector functions.^[33]^ The activation of β-adrenergic pathways potentiates these immunological perturbations through upregulation of proinflammatory gene expression, thereby promoting systemic subclinical inflammation.^[34]^ Studies have shown that sleep and inflammatory factors interact. Some cytokine levels increase when circadian rhythms are altered, and cytokines induce sleep.^[35]^ Changes in cytokines alter the inflammatory load, which affects sleep quality. Circadian misalignment upregulates IL-6 production, which can lead to RA progression.^[36]^ Additionally, C-reactive protein, an important indicator of RA activity assessment, is affected by sleep; sleep deprivation leads to elevated C-reactive protein concentrations.^[37]^

Sleep disorders disrupt circadian rhythmicity, leading to dysregulation of the hypothalamic-pituitary-adrenal axis and subsequent elevation of stress hormones, including cortisol. These neuroendocrine alterations have been shown to create a systemic pro-inflammatory state.^[32]^ Inflammatory cytokine release-mediated pathways are central to the pathogenesis of RA.^[38]^ Furthermore, there are synergies and interactions between inflammatory factors. For example, cytokines such as TNF and IL-1 can hypomethylate genes involved in RA pathogenesis, leading to increased expression of genes that cause the disease.^[39]^ TNF and IL-1 have been observed to induce IL-6 secretion by synovial fibroblasts, which in turn leads to CD4^+^ T-cell proliferation and the development of arthritis.^[40]^ Consequently, we hypothesize that sleep disorders establish an “inflammation-sleep disorder-RA” feedback loop by means of activating the immune-inflammatory pathway, as evidenced by PIV. While our study has demonstrated these associations, further research is necessary to elucidate the precise molecular mechanisms by which PIV is associated with trouble sleeping, to ascertain the temporal sequence of these interactions through longitudinal studies, and to identify potential therapeutic targets within this pathway.

Compared to previous PIV studies, this study had a larger sample size and more thorough control of confounding factors. A study from Turkey demonstrated that PIV, which may serve as a marker for complementary diagnostics, can be useful in detecting therapeutic efficacy and improving the prognosis of RA patients.^[41]^ PIV has the capacity to differentiate between active and inactive RA, thereby enabling the assessment of disease activity in patients.^[42]^ However, the methodological limitations of these studies are evident when one considers their single-center design and the relatively small number of participants. Emerging evidence highlights the clinical relevance of PIV as a prognostic biomarker in RA. The present study employed a more exhaustive sample, thereby ensuring the reliability of its conclusions. However, the impact of sleep disorders on RA has been found to vary in the results of previous studies. A study conducted in Saudi Arabia with a modest sample size demonstrated that sleep disorders were not associated with RA disease activity or patients’ quality of life.^[43]^ Conversely, another study demonstrated that poor sleep quality was associated with RA disease activity.^[44]^ Chinese cohort data identified short sleep duration as an independent risk factor for RA progression.^[18]^ A large-scale prospective Norwegian study identified an association between insomnia and subsequent RA onset.^[45]^ The observed discrepancy in outcomes may be attributable to variations in the samples utilized.

PIV is a routine blood data-derived indicator. It is highly accessible to healthcare professionals and can be widely utilized in clinical settings. Concurrently, the procedure is financially accessible to the patient, with minimal medical expenses and a diminished financial obligation. The employment of PIV has been demonstrated to facilitate the identification of individuals at elevated risk of RA during primary screening. Early intervention has been demonstrated to enhance the prognosis of RA patients, thereby reducing the incidence of joint damage and disability. The incorporation of sleep health as an intervenable factor into RA prevention and management strategies warrants further investigation. For clinicians, joint assessment of PIV and sleep status is more beneficial for RA patients.

The principal strength of this investigation lies in its extensive sample size, derived from a nationally representative dataset, incorporating demographic diversity across age, gender, racial/ethnic groups, and geographic distributions in the United States. To elucidate the relationship between PIV, trouble sleeping, and RA, as well as to adjust for potential confounding variables, logistic regression analyses were employed. RCS and smoothed curve fitting were used to explore the nonlinear relationship between PIV and RA. The present study has its own set of limitations. First, the cross-sectional design precludes the establishment of causal relationships between the examined variables. Second, the exposures and outcome relied on self-report; in particular, trouble sleeping was ascertained from a single self-reported question rather than a validated multi-item instrument or objective sleep assessment, and RA was defined by self-reported physician diagnosis. Third, the PIV calculation was based on single sampling, which may be influenced by transient status. Fourth, the study did not include potential confounders such as antirheumatic medication or psychological status.

In subsequent studies, the implementation of longitudinal or interventional research designs could be considered to validate causality, incorporate biomarkers or levels of inflammatory factors to further elucidate the mechanisms, explore the differential effects of different sleep types (insomnia, light sleep, nocturnal awakening, etc) on PIV and RA, and develop predictive models for early RA screening.

## 5. Conclusions

PIV and trouble sleeping have been shown to independently contribute to an elevated risk of RA. Our findings highlight a synergistic PIV-sleep interaction contributing to RA pathogenesis, distinct from their independent effects. Controlling inflammation in the body and lowering PIV levels, as well as ensuring a good night’s sleep, may help reduce the risk of RA. However, the confirmation of this causal relationship requires further prospective studies.

## Acknowledgments

We would like to thank all participants in this study.

## Author contributions

**Conceptualization:** Yuting Xiao, Jiayou Zhang, Shaoling Han, Lijuan Chen, Jing Wu.

**Data curation:** Yuting Xiao, Shaoling Han, Lijuan Chen, Junhui Liu, Jing Wu, Jing Su.

**Formal analysis:** Yuting Xiao.

**Methodology:** Yuting Xiao.

**Funding acquisition:** Jiayou Zhang.

**Project administration:** Jiayou Zhang.

**Supervision:** Jiayou Zhang, Lijuan Chen, Junhui Liu.

**Software:** Lijuan Chen.

**Validation:** Junhui Liu.

**Writing – original draft:** Yuting Xiao, Lijuan Chen, Jing Wu, Jing Su.

**Writing – review & editing:** Yuting Xiao, Jiayou Zhang, Shaoling Han, Jing Wu, Jing Su.
